# Supplementary figures and images for: DGAT1 and DGAT2 Inhibitors for Metabolic Dysfunction-Associated Steatotic Liver Disease (MASLD) Management: Benefits for Their Single or Combined Application
Source: Int J Mol Sci. 2024 Aug 21;25(16):9074. doi: 10.3390/ijms25169074 (PMC11354429; doi:10.3390/ijms25169074)

Figure S1

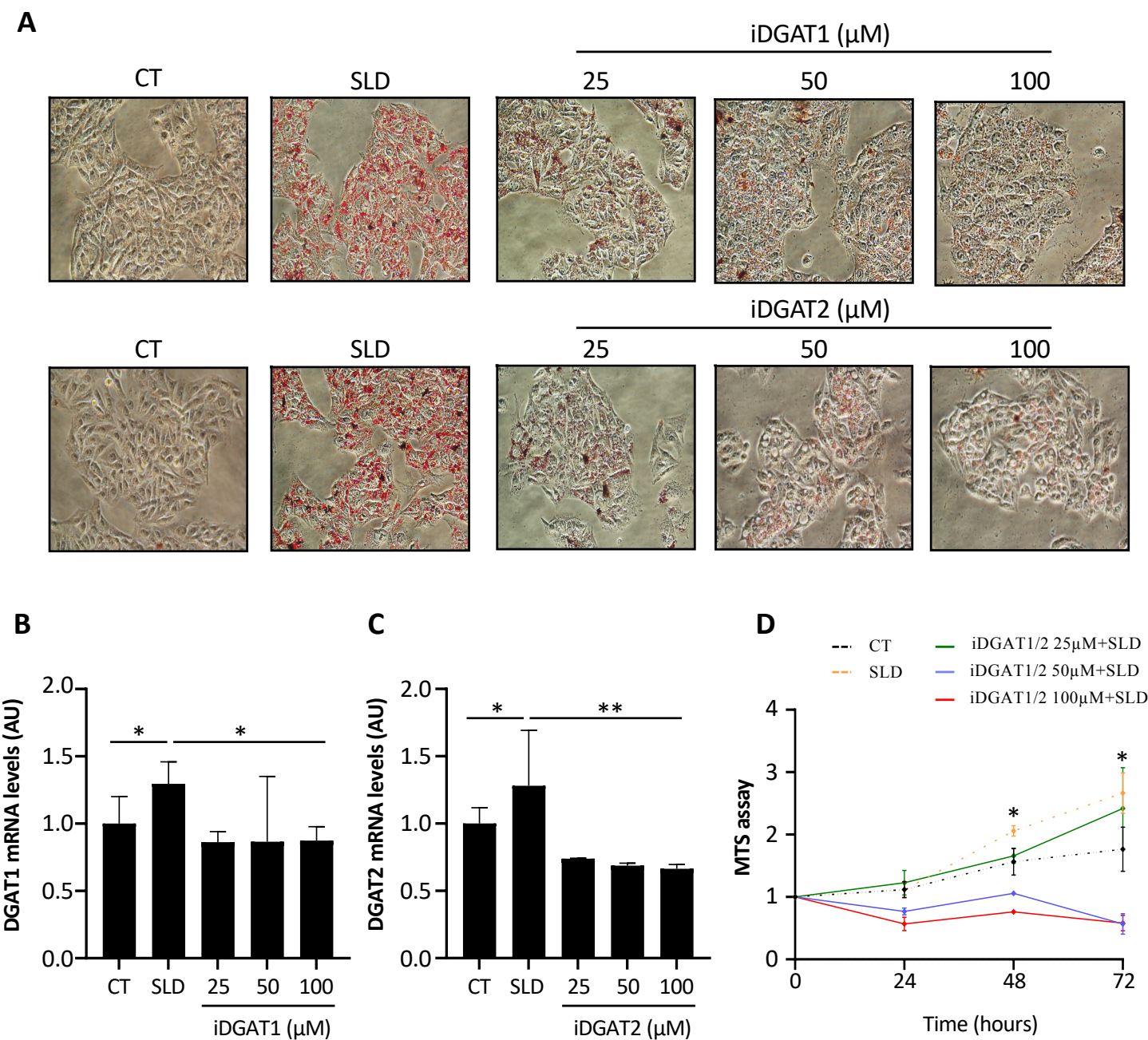

Figure S2

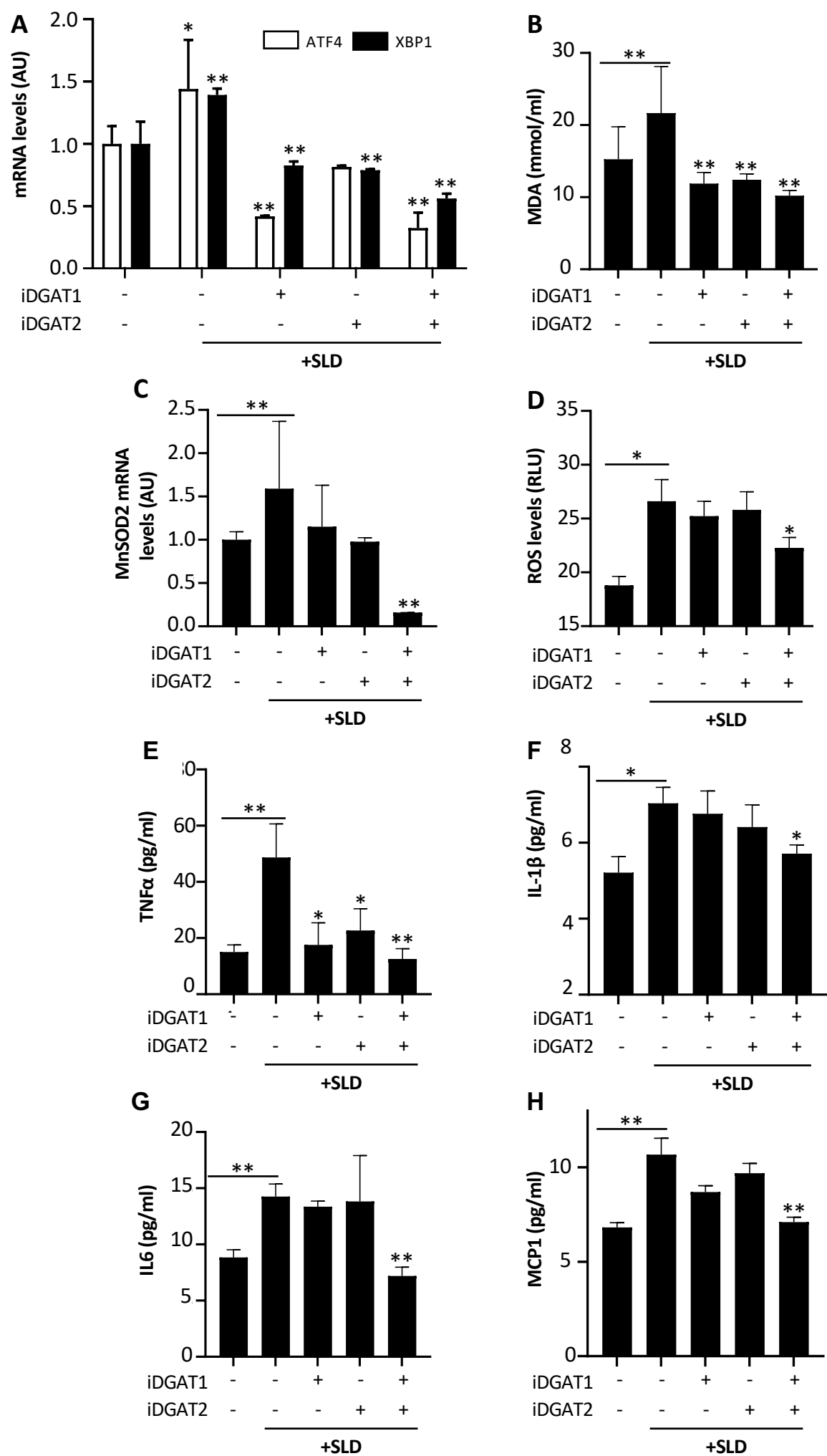

Figure S3

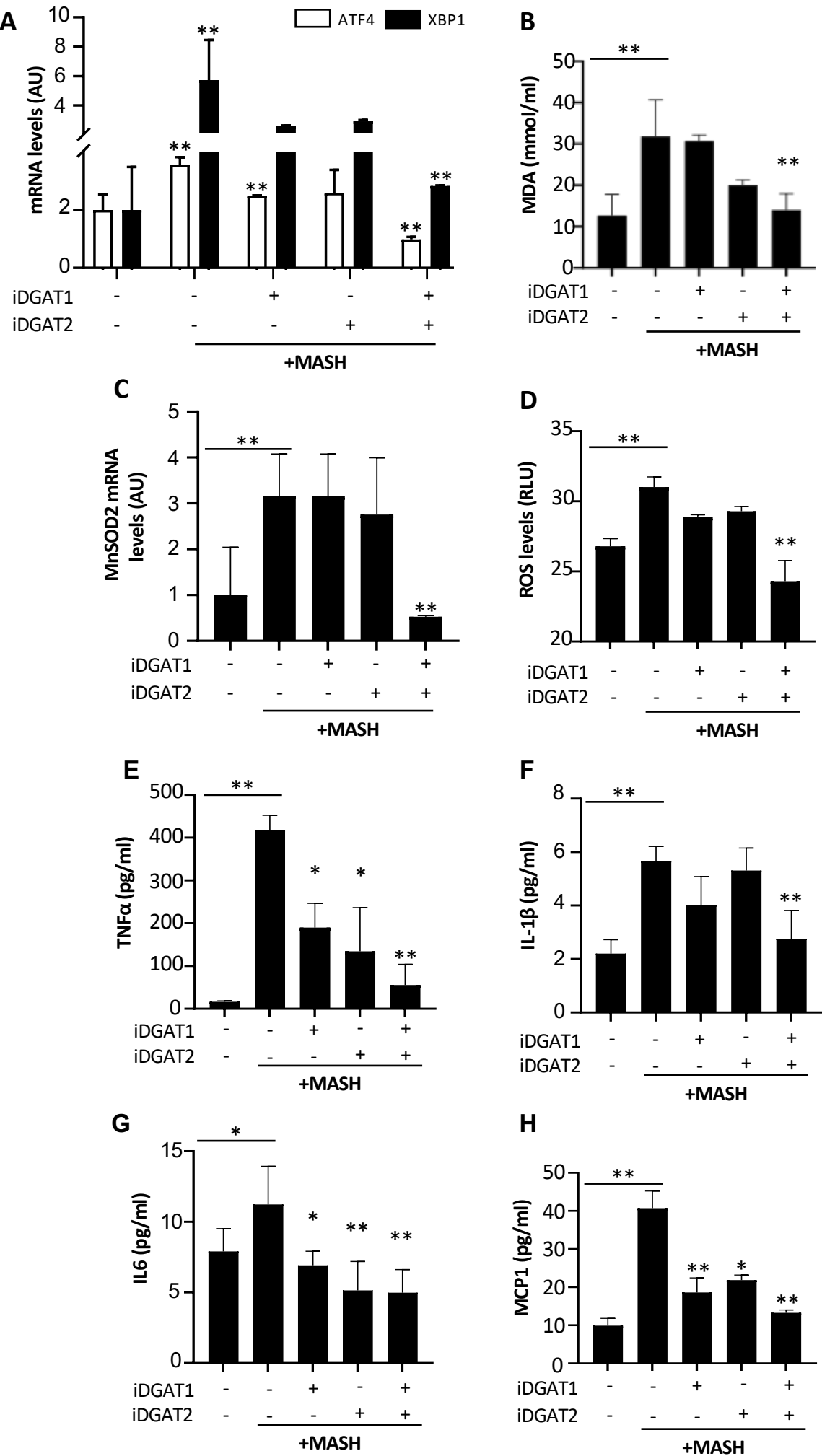

Figure S4

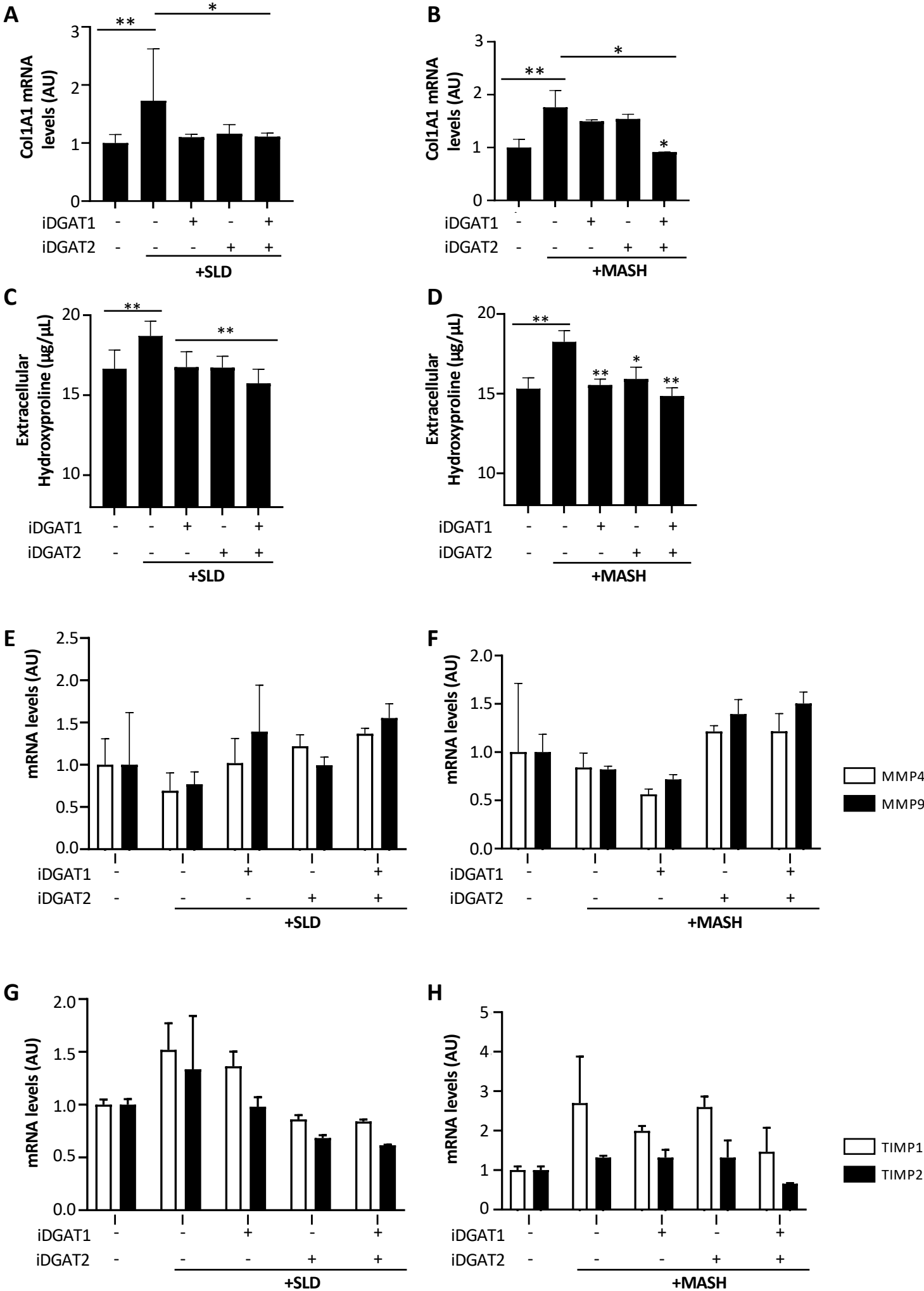

Supplement: Supplementary file 1 [file ijms-25-09074-s001.zip › ijms-3140190-Suppl Figures.pdf]
